# Analysis of swimming teaching programs, aquatic competencies and specific skills in school settings from early childhood to secondary education: A systematic review

**DOI:** 10.1371/journal.pone.0353477

**Published:** 2026-07-24

**Authors:** Alfonso Trinidad, Daniel Mendoza-Castejón, Laura Mercedes de la Calle, José Ángel del-Blanco-Muñiz

**Affiliations:** 1 Department of Education and Educational Innovation, Faculty of Law, Education and Humanities, Universidad Europea de Madrid, Villaviciosa de Odón, Madrid, Spain; 2 Aqualab Research Group, Universidad Europea de Madrid, Villaviciosa de Odón, Madrid, Spain; 3 Department of Sports Sciences, Faculty of Medicine, Health and Sports, Universidad Europea de Madrid, Villaviciosa de Odón, Madrid, Spain; 4 Department of Physiotherapy, Faculty of Medicine, Health and Sports, Universidad Europea de Madrid, Madrid, Spain; 5 Faculty of Medicine, Exercise and Cardiorespiratory Rehabilitation Research Group, Health and Sports, Universidad Europea de Madrid, Madrid, Spain; SPRINT - Sport Physical Activity and Health Research & Innovation Center, PORTUGAL

## Abstract

The teaching of swimming in school contexts has become increasingly important due to the benefits in the integral development of students and the prevention of aquatic accidents.This study analyses, by means of a systematic review, the aquatic teaching programmes and Competencies implemented in school settings over the last 25 years, from infant to secondary education. The Methodological approach was based on the PRISMA guidelines, with searches in scientific databases (PubMed, Scopus, Sport Discus and Web of Science) and secondary sources. Sixteen studies were selected according to the PICOS model, assessed with the STROBE and TREND tools. The results show a predominance of technical-utilitarian approaches focused on specific swimming skills, with little curricular integration and limited involvement of PE teachers. Most of the studies focused on infant and primary school, being scarce in secondary school. A high variability in duration, frequency, Contents and Assessment instruments was evidenced. The studies highlight the need for greater inclusion of swimming in school curricula, with more comprehensive pedagogical approaches adapted to each stage. It is concluded that although progress has been made, systematic implementation is still limited, requiring greater institutional support, teacher training and methodological coherence.

## Introduction

### The importance of swimming in childhood

Teaching swimming from an early age contributes to children’s comprehensive development for several reasons. Firstly, it serves as an important protective measure by improving water safety and reducing the risk of drowning, one of the leading causes of accidental death among children, particularly those under five years of age [1–3]. Furthermore, hybrid teaching models that combine pool and open-water instruction have been shown to improve water safety in children aged 7–11 by 50% to 80%. These approaches also enhance competencies related to buoyancy (37%) and general swimming ability (22%) through exposure to varied aquatic environments that promote adaptation and drowning prevention [4]. Secondly, swimming supports physical development by improving cardiovascular efficiency by up to 20% [5], while also enhancing respiratory musculature and increasing lung volume by 15% compared with other land-based sports [6,7]. In addition, the aquatic environment, which is approximately 800 times denser than air, facilitates high-resistance, low-impact muscle strengthening through continuous resistance during movement [8,9]. Swimming also improves motor coordination, enabling children to reach physical development milestones between 6 and 15 months earlier than average [10].

However, from these perspectives, swimming should not be understood exclusively as an exercise in survival or physical skill, as it also has a significant impact on children’s neuropsychological development. Psychologically, it reduces stress and anxiety [2], promoting emotional well-being through cortisol regulation [11]. In the social sphere, aquatic activities reinforce confidence and communication skills through interaction with peers and coaches [4]. Furthermore, the aquatic environment demands constant adaptation, which increases perceived self-efficacy and independence [12]. Therefore, empirical data associate swimming with a 20% increase in social competence and conflict resolution [13], consolidating it as a key tool for the acquisition of executive functions and adaptation to the school environment [14].

### The integration of swimming into the curriculum in different countries

The inclusion of aquatic content in the educational environment has been considered essential in school physical education, not only for its health benefits [15], but also for its ability to contribute to motor competency development in an environment with unique physical properties [16]. The scientific literature highlights that school teaching is the main vehicle for ensuring the concept of aquatic literacy, a term defined as the integration of the knowledge, skills, attitudes and behaviours necessary to interact safely and competently in aquatic environments [17,18], as it goes beyond simply “knowing how to swim”.

However, at the international level, its presence has often been limited to extracurricular activities or sports clubs [19–21] with uneven implementation in the formal curriculum. This scenario reflects a global pedagogical and challenge, with various countries attempting to transition from a technical model of “learning to swim” to a comprehensive aquatic competency approach, which is defined as “the multidimensional set of motor skills, safety knowledge and self-assessment abilities that enable a person to interact effectively and safely with the aquatic environment” [17]. As an illustrative example of this trend, in Spain there are two Royal Decrees [22,23] that allow the use of the aquatic environment for the development of student’s skills, but leave it’s incorporation to the discretion and autonomy of schools and physical education teachers. In this context, initiatives such as the “Swimming is Life” programme (Royal Spanish Swimming Federation) have emerged, which seeks to standardise teaching at the federation level. This situation contrasts with the reference frameworks of other countries such as England, France, Belgium and Australia, where swimming is compulsory within physical education for reasons of public safety and the promotion of active leisure [24–26]. However, regardless of the national model, there remains a global disconnect between curriculum guidelines and the practical reality of schools, highlighting the need for a more robust and standardised pedagogical approach in schools [27].

### Prospects and challenges for aquatic competencies in schools

One of the main challenges in teaching swimming at school age lies in the pedagogical approaches used, which are often based on utilitarian and mechanistic instruction programmes of a type. Although the first aquatic programmes aimed at children date back to the 1960s [28], scientific literature has documented their evolution through standardised programmes, especially in countries such as the United States and Canada [29]. These programmes, which have focused primarily on initiation and teaching core skills, safety in the aquatic environment and learning swimming styles [17,24,30], have mostly been designed by prestigious institutions such as World Aquatics (formerly FINA). As an illustrative example, the global initiative “Swimming for All – Swimming for Life” highlights the urgency of ensuring universal access to reduce drowning mortality globally [31]. Likewise, programmes promoted by national federations have also emerged, such as the aforementioned “Swimming is Life” or “Portugal Swimming” by the Portuguese Swimming Federation, along with other national programmes such as “Austswim” in Australia, “Savoir nager” in France, and the “National Curriculum for Swimming” in England and other Anglo-Saxon countries [32], as well as the Norwegian educational programme “Svømmedyktighet”.

Historically, most of these swimming teaching models have shown a marked inclination towards the systematic learning of sports techniques, prioritising the execution of competitive styles and mechanical performance [33]. This “sport oriented” approach limits children’s motor development by focusing on the repetition of rigid patterns, leaving their ability to respond to diverse environments in the background. In response to this, it is essential to adopt a developmental perspective that transcends traditional swimming styles, focusing on the acquisition of functional and comprehensive aquatic competence, which prepares children to interact safely with the environment [34]. However, current evidence also indicates that swimming skills alone are not sufficient to prevent drowning. Based on this premise, the European ALFAC (Aquatic Literacy for All Children) project [35] was launched, which aligns with the approaches of aquatic competence and aquatic literacy. Both concepts represent a paradigm shift that shifts the focus from isolated techniques and strokes to a broader approach that integrates physical, cognitive and affective competencies. Thus, the educational objective should shift from the pursuit of perfect technique to the development of comprehensive and utilitarian aquatic autonomy.

### The problem of implementing swimming

The incorporation of swimming in schools requires a multidisciplinary approach that goes beyond technical-utilitarian learning, understood as the acquisition of core aquatic skills so that children can achieve autonomy, safety and recreation in the environment, prioritising motor problem solving abilities rather than the rigid teaching of formal swimming styles [36]. In this vein, aquatic motor action is conceived as a tool for competency development and pedagogical dimensions that go beyond the traditional sports oriented view [37].

However, for this pedagogical vision to be effective and for the benefits of the practice to be consolidated, it would be essential to ensure the long term retention of skills [38]. In this regard, recent evidence suggests that the effectiveness of such retention depends on the methodological approach used. According to an exploratory review of motor learning methods [20], children learn through processes that range from instructional and linear models (based on ideal patterns and movement oriented feedback) to exploratory and non linear approaches, where learning is more individual and direct instruction is less prominent in order to avoid counterproductive effects. Currently, both perspectives are being applied through verbal instruction and repetition, also integrating emerging proposals through play, variability, and the use of everyday objects as teaching resources to make classes less predictable and more adaptive [39,40].

But the review by Minkels et al. [20] has limitations that restrict its pedagogical applicability, as it does not clarify whether the acquisition of skills took place during school hours (specifically in Physical Education or Psychomotor Skills) or whether the programmes were the result of extracurricular activities, classes in sports facilities or clubs outside the official curriculum. Furthermore, the study also lacks segmentation by educational stage (nursery, primary, secondary or sixth form) and has a limited sample range of ages from 5 to 12 years, without differentiating between levels of motor and curricular maturity. This lack of specificity regarding the educational context creates a gap in the scientific literature, which justifies the need for new systematic reviews that help to synthesise the evidence focused exclusively on the school environment and school hours, thus allowing the real impact of these methodologies within compulsory subjects to be analysed.

Among the main limitations to the implementation of these contents in the school environment are the availability of adequate facilities and the level of training of physical education teachers in relation to the aquatic environment, as well as their pedagogical preparation [41]. In many cases, teaching is outsourced to sports associations or institutions, whose professionals (monitors, instructors or technicians) adopt technical sports approaches that do not always conform to the pedagogical recommendations of the school context [42]. This situation raises a serious pedagogical debate, as technical staff possess aquatic skills due to federative training. However, unlike teachers, they lack university training in physical education and formal pedagogical competencies [43], which help to adapt the learning process to the individual requirements and skills of each student. Consequently, coaches are expected to focus their teaching on competitive performance or the acquisition of technical skills, while physical education teachers use their professional judgement to balance motor challenges with safety and actual aquatic competence [17,44], as well as aquatic literacy [45]. Furthermore, scientific literature has shown that there is no significant correlation between the technical qualifications of instructors and the actual progress of students, indicating that learning success depends more on pedagogical competence than on technical mastery [46]. Likewise, it has also been shown that students in technical programmes have deficiencies in transferring skills to real life risk situations, such as floating with clothes on, compared to those trained by teachers who prioritise comprehensive aquatic competence [17]. Furthermore, data indicate that teachers achieve greater inclusion and teaching effectiveness in heterogeneous groups thanks to their specific pedagogical training [43,44].

### Current developments in swimming instruction

The school environment can offer more varied and age appropriate motor experiences in stimulating contexts [47]. Schools, as promoters of physical activity, have begun to incorporate swimming into their school programmes [48], so that students can learn to swim and acquire water safety skills [49]. Furthermore, it has been observed that the earlier children start, the better they learn [35].

In this regard, some studies have observed improvements in aquatic skills after implementing short or médium term programmes [50]. However, in real school contexts, classes are usually weekly and short in duration, which raises doubts about their long term effectiveness. Therefore, it would be necessary to increase the frequency, intensity, duration, and variety of aquatic practice, not only to improve specific motor skills but also to consolidate general competencies. In this regard, it would be advisable to implement longer term interventions and analyse possible gender differences in this area [51].

Consequently, and considering the lack of evidence that clearly differentiates the impact of swimming within the formal curriculum from other sporting contexts, a synthesis that prioritises the school environment is essential. Therefore, the objective of this systematic review was to analyse the characteristics of aquatic interventions developed exclusively within school hours over the last 25 years, identifying the methodological approaches and action plans applied at different educational stages.

## Materials and methods

This systematic review was conducted following the updated PRISMA 2020 (Preferred Reporting Items for Systematic Reviews and Meta-Analyses) guidelines for transparent and reproducible reporting [52]. The review protocol was prospectively registered in the PROSPERO database (CRD420251071628).

### Search strategy

Published studies related to teaching swimming and aquatic skills through implementation programmes in school settings were identified through a systematic and comprehensive search of four electronic databases: PubMed, Scopus, SPORTDiscus, and Web of Science. To ensure comprehensive coverage, complementary sources such as Google Scholar and manual review of reference lists were also included.

The search strategy was developed using a structured approach, combining controlled vocabulary (e.g., MeSH terms in PubMed such as *“Swimming” [Mesh]*, *“Schools” [Mesh]*, *“Physical education and training” [Mesh]*) and free text terms to maximise sensitivity and capture studies that might not be indexed under specific subject headings.

The strategy combined three conceptual clusters: (1) aquatic activities and skills (e.g., “swimming”, “aquatic skills”, “water safety”, “aquatic competence”), (2) educational context (e.g., “school”, “physical education”, “syllabus”, “schoolchildren) and (3) educational interventions or outcomes (e.g., “teaching programme”, “instruction”, “pedagogical approach”, “learning outcomes”). These groups were connected using Boolean operators (OR within groups; AND between groups).

Searches were adapted to the syntax of each database (e.g., [tiab] in PubMed, TITLE-ABS-KEY in Scopus) and limited to titles and abstracts to ensure relevance. The complete Boolean queries including the lists of terms and operators are listed in section supporting information (S1 File. Search Strategy).

### Eligibility criteria

Original scientific research related to the teaching of swimming and aquatic skills in the school environment and during school hours was considered. Selected studies had to be published in English or Spanish between January 2000 and December 2025 in peer-reviewed journals with an impact factor.

To assess the quality of the reports, the STROBE (for observational studies) and TREND (for non-randomised intervention studies) checklists were applied. These tools assess the completeness and transparency of the reports, but not the methodological quality or risk of bias. Each item on the checklist was scored as “reported” (1) or “not reported” (0), and the total percentage compliance was calculated. Only studies with at least 50% compliance with the relevant checklist were included. This threshold was adopted to ensure a minimum level of completeness in reporting, while allowing for the inclusion of studies of various designs and publication formats, as recommended in previous methodological reviews [53].

Following the PICOS model (population, interventions, comparisons, outcomes, and study design) [54], the inclusion criteria were: (1) Population: school-aged students (children, primary, secondary, or high school); (2) Interventions: educational programmes carried out during school hours and taught by teachers or external staff; (3) Comparisons: comparison of variables related to swimming between control and/or experimental groups (for experimental or quasi-experimental designs); (4) Outcomes: variables related to swimming contents and aquatic skills (e.g., techniques, activities, tasks); (5) Study design: descriptive, observational, experimental, and longitudinal studies.

The exclusion criteria were as follows: (1) aquatic programmes conducted outside the school context (e.g., extracurricular activities or activities not related to physical education); (2) studies that did not involve an educational stage (children, primary school, secondary school, or high school); (3) studies whose samples did not include school-aged students or included students with disabilities or diagnosed disorders; (4) studies focused on swimming training rather than teaching or learning processes.

### Data extraction strategy

To ensure the reliability of the selection process and the eligibility of the included studies, two authors (José Ángeldel-Blanco-Muñiz and Daniel Mendoza-Castejón) independently screened the records according to the PRISMA guidelines for systematic reviews [55]. Agreement was required for the inclusion of studies. In case of disagreement, a third reviewer (Alfonso Trinidad) was consulted to reach a consensus. The review protocol was carried out between November and December 2024, and on 1 December 2025 an updated search was performed using the same methods as in the initial search.

The research team developed a structured ad hoc data extraction form based on the review objectives and the PICOS framework. The form was tested independently by two reviewers on a random sample of five included studies to assess its clarity, consistency, and completeness. Minor adjustments were made after the pilot phase.

Subsequently, data extraction was performed independently by the same two reviewers. Inter-rater reliability was assessed using Cohen’s kappa coefficient, which yielded a value of 0.87, indicating near-perfect agreement between reviewers. Discrepancies identified during the extraction process were resolved through discussion and consensus, with the involvement of a third reviewer when necessary.

The variables extracted included: (a) author(s), year of publication, country, sample characteristics (size, gender, age), educational level, and study objectives; (b) study design, duration of intervention, instruments and materials used; and (c) curriculum, physical, sports, and recreational activities, grouping of aquatic tasks/exercises, and type of aquatic approach.

The final version of the data extraction form is available upon reasonable request to ensure transparency and replicability.

### Classification criteria and coding procedure

During data extraction, two key analytical categories were applied: instructional activities and aquatic approaches. These categories were defined a priori based on previous bibliography on physical education and aquatic pedagogy, and were implemented using a structured coding rubric.

a) Instructional activities refer to specific teaching tasks, exercises, or learning sequences implemented during the intervention. These include both individual and group activities aimed at competency development in the aquatic domain, motor skills, or specific swimming techniques. Activities were coded by type (e.g., exercises, games, circuits), modality (e.g., guided, free practice), and objective (technical, cognitive, social).
b) Aquatic approaches refer to the general pedagogical philosophy or framework that guided the intervention. Two main approaches were used to classify the studies:
  - “Technical-functional orientation” referred to studies primarily focused on the development of functional aquatic competencies, such as swimming technique, breathing control, balance, water safety, or survival skills.
  - “Educational-curricular orientation” referred to studies that explicitly linked aquatic activities with broader educational aims and curriculum-related competencies within school contexts, including students’ holistic development, Physical Education learning objectives, inclusion, social interaction, or health education.

The rubric used for classification included observable indicators such as lesson structure, stated objectives, assessment criteria, and references to educational models. Two reviewers coded each study independently, and discrepancies were resolved through debates. The rubric is available upon request to ensure transparency and replicability.

## Results

### Review statistics

**Fig 1:**
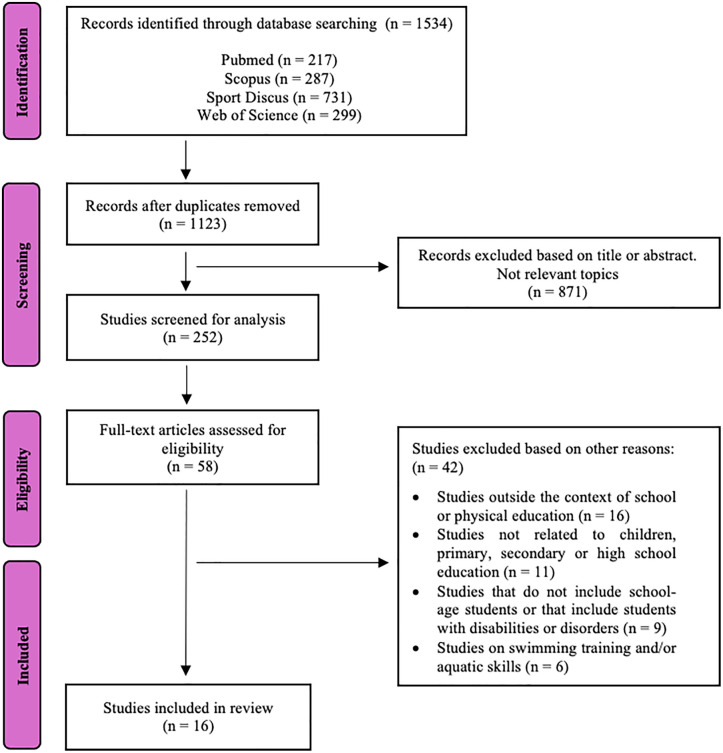
Flow diagram for screening and selection of studies according to preferred reporting item for systematic reviews and analysis (PRISMA).

The review included a total of 16 articles. The selection process took into account the guidelines set by PRISMA. A total of 1534 scientific studies were identified in the four digital databases. Subsequently, duplicate files were eliminated and those that were not relevant were excluded through a preliminary reading of the degree and abstract (1123). Next, full-text studies that did not meet the eligibility criteria were removed (42). Finally, the remaining studies based on swimming and aquatic skills implementation programmes were considered (16) (Fig 1).

### Assessment of the quality of the reports of the included studies

The STROBE checklist was used to assess the reporting quality of selected observational studies [56]. This checklist consists of 22 items grouped into six categories pertaining to the different sections of the study: Title-Abstract (item 1), Introduction (items 2 and 3), Methods (items 4–12), Results (items 13–17), Discussion (items 18–21) and Funding (item 22). A score of “0” was assigned to items that were incomplete or lacked information, and “1” to items that were accurately described. The overall grade obtained from the sum of the item values was categorised according to the following levels: very low quality (0–4 points); low quality (5–8 points); medium quality (9–12 points); high quality (13–16 points); and very high quality (17–22 points).

In our review, 75% of the observational studies were of moderate to low quality (50–60%), while 25% were of moderate quality (>60%). The assessment of quality reporting according to the STROBE list in included observational studies is shown in Table 1 below.

**Table 1:** Assessment of quality reporting according to the STROBE list of included observational studies.

Author(s) and year of publication	STROBE score
Moreno-Murcia & Ruíz-Pérez [57]	50%
Bielec [58]	51,7%
Martins et al. [59]	56,8%
Chan et al. [60]	61,4%
Strasylova et al. [61]	50,9%
Moreno-Murcia et al. [62]	65,9%
Pratt et al. [63]	54,5%
Sundan et al. [64]	50,6%

To complement the assessment of reporting quality of the included experimental studies, especially those with quasi-experimental or non randomised design, the TREND guide was taken into account. This tool, developed by Des Jarlais et al. [65], proposes a 22 item checklist designed to improve transparency and rigour in the presentation of intervention studies in which participants were not randomly assigned. Its use is particularly relevant in applied research in educational and public health contexts, such as aquatic education programmes in school settings, where randomised designs may not be feasible or ethical. The application of the TREND guide allows for the assessment of key aspects of design, implementation, analysis and the introduction of results, thus ensuring a more critical and reliable interpretation of the findings.

In our review, 75% of the experimental studies were of moderate to low methodological quality (50–60%), while 25% were of moderate quality (>60%). The assessment of quality reporting according to the TREND checklist in non-randomised interventional studies is shown in Table 2 below.

**Table 2:** Assessment of quality reporting according to the TREND list in non-randomised interventional studies.

Author(s) and year of publication	TREND score
Jurak et al. [66]	50,2%
Karatrantou et al. [67]	63,6%
Stankovic et al. [68]	52,3%
Moura et al. [69]	54,5%
Moura et al. [70]	50%
Moreno-Murcia et al. [71]	65,9%
Pratt et al. [72]	52,3%
Shen et al. [73]	56,8%

### Risk of bias in included studies

The risk of bias of the included studies was assessed using two tools based on study design. For observational studies (n = 8), an adapted version of the Newcastle–Ottawa Scale (NOS) was applied, yielding scores between 5 and 6 out of 9. All studies were categorized as having a moderate risk of bias, primarily due to lack of adjustment for confounders and insufficient reporting of participant recruitment (Table 3).

**Table 3:** Newcastle-Otawa scale (NOS).

Study	Selection (max 4)	Comparability (max 2)	Outcome/Exposure (max 3)	Total (max 9)	Risk of bias
Moreno-Murcia & Ruiz-Pérez [57]	3	0	3	6	Moderate risk of bias
Bielec [58]	3	0	3	6	Moderate risk of bias
Martins et al. [59]	3	0	3	6	Moderate risk of bias
Chan et al. [60]	3	0	3	6	Moderate risk of bias
Stanković et al. [68]	3	0	3	6	Moderate risk of bias
Strašilová et al. [61]	3	0	3	6	Moderate risk of bias
Pratt et al. [63]	3	0	3	6	Moderate risk of bias
Sundan et al. [64]	3	0	2	5	Moderate risk of bias

**Fig 2:**
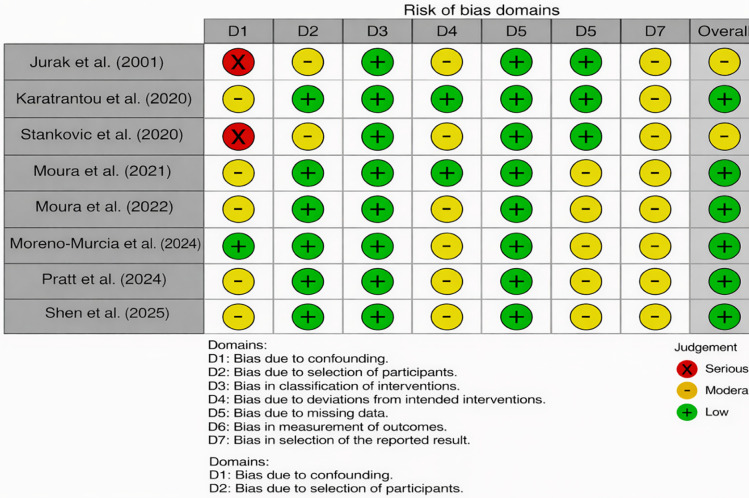
Robins-I.

For quasi-experimental studies (n = 8), the ROBINS-I tool was applied to assess risk of bias across seven domains. The overall risk of bias was judged to be low in the majority of studies (6 out of 8), while two studies [66,68] were rated as having a moderate overall risk. The domains with consistently low risk across studies included classification of interventions (D3), missing data (D5), and measurement of outcomes (D6). Conversely, bias due to confounding (D1) was the domain with the highest concern, with two studies presenting a serious risk and the rest rated as moderate. Additionally, moderate risk was frequently observed in selection of reported results (D7) and deviations from intended interventions (D4). Importantly, no study was judged to be at critical risk of bias in any domain. A domain-specific summary is shown in Fig 2.

### Characteristics of the studies reviewed

Regarding the geographical origin of the scientific output identified between 2001 and 2025 (n = 16), 75% of the studies (n = 12) were located in Europe [57–59,61–64,66–68,71,72], while 12.5% (n = 2) corresponded to the American continent [69,70] and another 12.5% (n = 2) to the Asian context [60,73]. In terms of distribution by country, Spain had the highest frequency of publications with 18.7%, followed by Brazil, China, and England, with 12.5% each.

In relation to the characteristics of the participants, the samples analysed were aged between 3 and 14 years old and were predominantly heterogeneous, except in three research studies in which this condition was not specified [60,66,68]. In terms of educational stage, primary education accounted for 68.7% of the output with eleven studies [59–61,63,64,66–70,72] In addition, preschool education accounted for 25% of the scientific evidence [57,62,71,73], while secondary education accounted for 6.2% with only one study [58].

Finally, the research objectives were mainly oriented towards the validation of measurement tools (such as scales or questionnaires) and the implementation of teaching programmes. Regarding the achievement of these objectives, the results consistently indicated the effectiveness of interventions for the acquisition of immediate motor skills. However, technical teaching prevailed over comprehensive aquatic competence for drowning prevention, as did the absence of longitudinal assessments in the programmes analysed.

Table 4 details the key characteristics of the studies included in this research.

**Table 4:** Main descriptive characteristics of the included studies.

Author(s)	Country	Sample	Age/ Course	Educational level	Aim(s) of the study
Jurak et al. [66]	Slovenia	370 childrens	8-9	Primary	To develop two experimental swimming teaching programmes and compare them with an established swimming programme in a Swimming School.
Moreno-Murcia and Ruíz-Pérez [57]	Spain	100 (46 boys; 54 girls)	4.5 ± 0.67	Children	To develop and validate a scale to measure children’s perceived motor competence in water.
Bielec [58]	Poland	116 (60 boys; 56 girls)	14.0 ± 0.3	Secondary	To find out the opinion of secondary school pupils on the content of swimming lessons given as part of Compulsory Physical Education classes.
Martins et al. [59]	Portugal	140 (84 boys; 56 girls)	1st- 4th	Primary	To describe the level of global motor development in primary school with and without previous swimming practice.
Chan et al. [60]	China	1614 childrens	6.40 ± 0.52	Primary	Assessment of the reliability and validity of the Swimming Competency Questionnaire (SCQ) in primary school through a cross-curricular survey and a pre-posttest quasi-experiment for learning to swim.
Karatrantou et al. [67]	Greece	326 (179 boys; 147 girls)	8-9	Primary	To examine the test-retest reliability of the aquatics test. To research the effectiveness of swimming lessons during the physical education year on aquatics. To examine the enjoyment of swimming class in primary 3rd^(er)^ grade.
Moreno-Murcia et al. [62]	Spain	384 (195 boys; 189 girls) 444 (235 boys; 208 girls)	4.02 ± 0.82 4.45 ± 0.84	Children	To design and analyse the validity of the Scale for Measuring Aquatic Competence in Children (SMACC) to assess aquatic competence in children. To verify the relationship between actual competence obtained with the SMACC and perceived aquatic competence, as well as its differences according to gender and age.
Strašilová et al. [61]	Czech Republic	14793 childrens	2º	Primary	Assessment of swimming skills of primary school pupils during the years 1995–2015.
Stanković et al. [58]	Serbia	100 childrens	11 ± 6	Primary	To examine and compare the effects of physical education (PE) lessons with additional swimming training, in relation to regular PE teaching, on the motor abilities of younger school-age pupils.
Moura et al. [69]	Brazil	31 (16 boys; 15 girls)	7-9	Primary	To verify the impact of two swimming learning programmes on aquatic readiness and motor coordination at school age.
Pratt et al. [63]	England	201 (96 boys, 105 girls)	7.8 ± 0.63	Primary	To develop a valid and reliable Assessment method to analyse aquatic motor competence, the “Aquatic Movement Protocol (AMP).” To research the associations between land and water movements to determine whether swimming has a positive impact on land motor competence.
Moura et al. [70]	Brazil	50 (24 boys; 26 girls)	8.34 ± 1.10	Primary	To verify the effects of swimming lessons on aquatic skills and motor coordination.
Moreno-Murcia et al. [71]	Spain	385 (195 boys; 189 girls)	3-5	Children	To show how a guided discovery-based teaching method would affect the teaching of children’s aquatic competence in schoolchildren with different levels of competence.
Pratt et al. [72]	England	107 (52 boys, 55 girls)	7.8 ± 0.63	Primary	To carry out a comprehensive assessment of the children’s actual swimming level according to the Norwegian standard defined by the curricular objectives for physical education in primary school.
Sundan et al. [64]	Norway	2421 (1234 boys; 1187 girls)	9-10	Primary	To examine the effects of a swimming intervention on children’s motor competence.
Shen et al. [73]	China	31 (16 boys; 15 girls)	5-6	Children	Assessment of the impact of a swimming intervention on motor skills competencies at preschool age.

### Methodological analysis of the studies reviewed

Of the sixteen studies selected, eight presented an observational research design [57–64], while the remaining eight studies were quasi-experimental [66–73].

In relation to intervention time, there is a great deal of variability among the research reviewed, both in the number and length of sessions and in the total duration of the intervention. Two of the sixteen studies reviewed had less than 10 sessions [67,72], while four studies had between 10 and 16 sessions [66,69–71], but only four studies had between 20 and 24 sessions [57,60,68,73]. In contrast, five studies did not specify the number of sessions performed [58,59,62–64].

There were also differences in session duration times. Although the sixteen studies were analysed strictly based on the information provided by the authors, without assumptions or imputation of data for incomplete records. Only two studies used 50 minutes per session [69,70] and two others used 90 and 180 minutes [66,67]. However, in the rest of the studies, and in the majority of them, the time devoted to each session was 45 minutes [64,67,68,] and 60 minutes [57,58,60,66,72,73]. In contrast, four other studies did not specify the duration in minutes of each session [59,62,63,71]. Finally, the total duration of the interventions in the sixteen studies ranged from 2 to 16 weeks, although they were analysed qualitatively when specific session counts or durations were missing to avoid bias in the calculation of the total intensity of the intervention. However, only two studies did not specify this data [59] or were longitudinal studies [61]. This approach ensured the representativeness of the total sample (n = 16), despite the heterogeneity and lack of detailed information in some of the sources analysed.

With regard to the variables and measurement instruments reviewed, the results revealed a methodological gap and a conceptual discrepancy with current pedagogical trends. Only five of the sixteen studies used standard, pictorial, and dimensional scales (socio-affective, cognitive, motor) to assess aquatic competence and swimming through different assessable items [57,61,62,64,66]. Four other studies used different questionnaires to measure attitudes towards swimming [58], intrinsic motivation [67] and aquatic competence [60], as well as fear of drowning and swimming opportunities [72], with no standardised instruments identified to assess teacher readiness or student participation. Two studies used instruction protocols for aquatic skills and movements [63,72] and only one used a test with different swimming tasks [67]. Two studies also used tests to measure motor coordination, combined with an observational checklist of different aquatic elements [69,70], based on motor development models from previous decades [e.g.,75]. Only one study used a test to measure gross motor development [59]. However, two other studies used the standardised Eurofit [68] and Movement Assessment for Children (MABC-2) [73] batteries to assess overall motor competence, rather than focusing solely on comprehensive aquatic literacy. Furthermore, only one study used a skill recording system [61], while another used a behaviour assessment rubric [71]. Furthermore, only three of the sixteen studies used complementary materials in their interventions (mainly boards, noodles and other flotation devices) [66,67,73], as the rest did not specify the use of any type of material. However, in general, most of the tools identified focused on technical execution and swimming strokes, with fewer instruments aligned with functional aquatic competence, which limited the comprehensive assessment of aquatic literacy by omitting critical dimensions such as risk management and adaptability to the environment.

In summary, the analysis revealed considerable heterogeneity in the design of the interventions, which ranged in duration from 2 to 16 weeks. This variability in duration and frequency highlighted the absence of a unified methodological standard for teaching swimming in schools. The scope of the objectives seemed to be influenced by time, as extensive programmes (12–16 weeks) focused on technical efficiency and style mastery, while short programmes (2–5 weeks) were limited to initial adaptation and core skills. In contrast, the description of auxiliary material was minimal (18.7%), suggesting that these resources were not considered control variables in the designs analysed, which compromises the replicability of the protocols.

Table 5 presents the key methodological characteristics of the studies included in this research.

**Table 5:** Main methodological characteristics of the included studies.

Author(s)	Study design	Intervention time	Instrument(s)	Auxiliary material used
Jurak et al. [66]	Experimental	Study 1 10 and 15 sessions 60 and 90 min 10 weeks Study 2 15 sessions 90 min 10 weeks	5-level scale with official standards *(Jurak et al., 1998)*	Boards, balls, buoys, air mattresses, slides, rings, floating poles... Sinkables, underwater tunnels, buoys, balls, baskets, anchors, balloons, cartoons and music.
Moreno-Murcia and Ruíz-Pérez [57]	Observational	20 sessions 60 min 1day/week 20 weeks	Pictorial Scale of Competencies Aquatic Perceived Competence Scale (APCPS)	Not specified
Bielec [58]	Observational	60 min 1day/week	Diagnostic questionnaire *(Pilch & Bauman, 2005)*	Not specified
Martins et al. [59]	Observational	Not specified	Test of Gross Motor Development 2 *(Ulrich and Sanford, 2000)*	Not specified
Chan et al. [60]	Observational	20 sessions 60 min	Swimming Competencies Questionnaire (SCQ) Competence Questionnaire (SCQ)	Not specified
Karatrantou et al. [67]	Experimental	Study 1 2 sessions 180 min 1 day/week 2 weeks Study 2 9 sessions 45 min 1 day/week 9 weeks	Study 1 and 2 Swimming test protocol *(Varveri et al., 2016)* Study 3 Intrinsic motivation questionnaire *(McAuley et al., 1989)*	Study 2 Boards, noodles, seat belts...
Moreno-Murcia et al. [62]	Observational	1day/week	Scale for Measuring Aquatic Competencies Aquatic Competence in Children (SMACC) Pictorial Scale of Perceived Aquatic Competence (PSPAP) Perceived Aquatic Proficiency Scale (PSPAP) *(Moreno-Murcia and Ruiz-Pérez, 2008)*	Not specified
Strašilová et al. [61]	Observational	20 years	Skill register	Not specified
Stanković et al. [68]	Experimental	24 sessions 45 min 2 days/week 12 weeks	Standardised Eurofit battery	Not specified
Moura et al. [69]	Experimental	12 sessions 50 min 1 day/week 12 weeks	Observational checklist *(Langerdorfer & Bruya, 1995)* Body co-ordination test (KTK) *(Kiphard & Schilling, 1974)*	Not specified
Pratt et al. [63]	Observational	Not specified	Aquatic Movement Protocol (AMP)	Not specified
Moura et al. [70]	Experimental	12 sessions 50 min 1 day/week 12 weeks	Observational checklist *(Langerdorfer & Bruya, 1995)* Body co-ordination test (KTK) *(Kiphard & Schilling, 1974)*	Not specified
Moreno-Murcia et al. [71]	Experimental	16 sessions 1 day/week 16 months	Aquatic Competence Scale (SMACC) *(Moreno-Murcia et al., 2020)* Behavioural Assessment Rubric	Not specified
Pratt et al. [72]	Experimental	6 sessions 60 min 1 day/week 6 weeks	Aquatic Movement Protocol (AMP) Drowning Fear Questionnaire and swimming opportunities	Not specified
Sundan et al. [64]	Observational	45 min 6-32 weeks	Swimming Competencies Assessment Scale (SCAS) Swimming Proficiency Assessment Scale (SCAS) *(Sundan et al., 2023)*	Not specified
Shen et al. [73]	Experimental	60 min 20 sessions 3 days/week 7 weeks	Movement Assessment Battery Movement Assessment Battery (MABC-2)	Noodle, plank, back float...

### A plan for activities that are instructional, physical and recreational of the studies reviewed

In the sixteen studies reviewed there were different syllabus for instructional, physical-sport and recreational activities, related to the teaching of swimming and water skills. These plans were grouped by types of aquatic tasks and/or exercises, and by aquatic approaches or purposes, due to the great variety of practical proposals made and found in the literature.

Twelve of the sixteen studies carried out aquatic activities involving static and dynamic floating, together with breathing exercises [57,60–64,67–70,72,73]. While ten studies performed activities related to pool jumping Skill [57,58,63,64,66,68–73] and seven studies activities linked to pulses and water slides [60,63,68–70,72,73]

In relation to aquatic movements, eight studies proposed basic tasks and/or exercises [57,59,61,62,64,67,68,71], while only three studies proposed complex shifts in their interventions [63,66,73]. Furthermore, only five studies combined basic and complex movements in their proposed aquatic tasks and/or exercises [58,60,69,70,72].

On the other hand, eleven studies employed immersion exercises [58,60–64,67–70,72]. On the other hand, only four studies conducted aquatic games during the practice sessions [57,66,68,69] and only two studies performed manipulation activities, throws and catches in the water [57,73], proprioception work [63,72] and dry exercises before water entry [60,68].

In addition, regarding the pedagogical orientation of the aquatic activities, twelve studies primarily adopted a technical-functional orientation focused on the development of functional aquatic competencies through specific aquatic tasks and exercises, such as swimming technique, breathing control, balance, water safety, or survival skills [57–63,66,67,69,70,73]. In contrast, four studies explicitly linked aquatic activities to broader educational aims and curriculum-related competencies within school contexts [64,68,71,72]. In these studies, aquatic activities were presented not only as a means of acquiring aquatic skills, but also as pedagogical tools contributing to students’ holistic development, Physical Education learning objectives, social interaction, inclusion, and health education.

In summary, analysis of the relationship between the contents and pedagogical approaches of the programmes implemented revealed that the technical-utilitarian model prevails over the educational-curricular model (75% versus 25%). Although 75% of the programmes included flotation and breathing exercises, the focus was on propulsion and technical styles. Only 25% of the studies analysed linked aquatic activity to the objectives of the physical education Syllabus. Furthermore, comparing these data with the year of publication did not identify any temporal trend towards more educational approaches. The technical model has continued to prevail in studies from the early 2000s as well as those published around 2020. This stability in frequencies indicates a lack of evolution in methodology, as the technical approach has remained dominant in school swimming over the last two decades.

Table 6 shows the programme of instructional, physical and recreational activities in the studies included in this research.

**Table 6:** Syllabus of instructional, physical-sports and recreational activities of the studies included in the review.

Author(s)	Instructional, physical-sport and recreational activity plans	Grouping of aquatic tasks/exercises	Pedagogical orientation of the aquatic activity*
Jurak et al. [66]	Breaststroke technique exercises Games, races, relays and additional group tasks Distraction games Aquatic jumps	Complex movements Aquatic games Jumping	Technical-functional orientation
Moreno-Murcia & Ruíz-Pérez [57]	Floating Propulsion Manipulation Balance Breathing control Water entry (individual and with equipment) Group games without support	Static and dynamic floating Core basic movements Handling, Throwing and Catching Breathing Jumping Aquatic games	Technical-functional orientation
Bielec [58]	Swimming with your legs Swimming with arms Full swim Jumping into the water Jumping into the water standing up Diving and searching for objects	Core and complex movements Diving Jumping	Technical-functional orientation
Martins et al. [59]	Aquatic Utility Skills	Core movements	Technical-functional orientation
Chan et al. [60]	Warm-up and preparation (in pool/out of pool) Early practice: • Aqua walking • Holding breath underwater • Breathing exercise • Floating and stationary exercise • Gliding exercise Front-crawl kicking practice: • Poolside kicking (sitting position) • Poolside kicking (holding the pool edge, head up) • Poolside kicking (head down, holding breath) • Poolside kicking (head up only for breathing) • Near poolside kicking (arms pushed away from the edge and return) • Front-crawl kicking (jump start from the bottom of the pool) • Front-crawl kicking (jump start from the pool wall) • Kickboard kicking (head up) • Kickboard kicking (head down, holding breath) • Kickboard kicking (head up only for breathing) • Kickboard kicking (holding kickboard with single arm) Front-crawl pulling practice: • Pulling on the ground (single-arm and alternate-arm) • Pulling whilst standing in the pool (single-arm and alternate-arm) • Single-arm pulling with poolside kicking (head down, holding breath) • Single-arm pulling with poolside kicking drills (head up only for breathing) • Alternate-arm pulling with poolside kicking drills (head down, holding breath) • Alternate-arm pulling with poolside kicking drills (head up) • Alternate-arm pulling with poolside kicking drills (head down, holding breath) • Alternate-arm pulling with poolside kicking drills (head up only for breathing) Alternate-arm pulling with kicking • Alternate-arm pulling with pull buoy: ◦ Front-crawl pulling with forward movement assistance Coordination of kicking and pulling: • Single-arm pulling with kickboard kicking (head down, holding breath) • Single-arm pulling with kickboard kicking (head up only for breathing) • Alternate-arm pulling with kickboard kicking (head down, holding breath) • Alternate-arm pulling with kickboard kicking (head up only for breathing) • Front-crawl (head down, holding breath) • Front-crawl (head up only for breathing) Front-crawl technique improvement	Static and dynamic buoyancy Breathing Diving Impulses and slides Core and complex movements Dryland Workout	Technical-functional orientation
Karatrantou et al. [67]	Buoyancy Balance Breathing control Free swimming technique Underwater vision Underwater hearing Underwater apnoea Exhalation diving	Static and dynamic buoyancy Breathing Core basic movements Diving	Technical-functional orientation
Moreno-Murcia et al. [62]	Breathing Dorsal Balance Manipulation Ventral and dorsal movement Turns Immersion	Static buoyancy Breathing Immersion Turns Core movements	Technical-functional orientation
Strašilová et al. [61]	Diving (face and head) Floating on the belly Float on back Swimming in shallow water (5 m) Swimming in deep water (+ 5 m)	Dive Static and dynamic buoyancy Core movements	Technical-functional orientation
Stanković et al. [68]	Dry exercises Breathing exercises Underwater gaze exercises Getting used to being in the water Horizontal position exercises Water sliding exercises Water games and diving Jumps and basic swimming core techniques	Dryland Workout Breathing Diving Thrusts and glides Core basic movements Water games Jumps	Educational-curricular orientation
Moura et al. [69]	Water entry Water orientation and adjustment at vertical position Breath control (immersion of the face and eye-opening) Horizontal buoyancy Body position at ventral gliding Body position at dorsal gliding Body position at longitudinal rotation in gliding Body position at front and back somersaults Leg kicking with breath control at ventral body position, with flutter boards Without any flutter device Egg kicking with breath control at dorsal body position with flutter boards Without any flutter device Feet-first entry Head-first entry Autonomy in a deep pool (legs and arms displacement) Vertical buoyancy at deep water Deep water immersion	Static buoyancy Breathing Immersion Thrusts and slides Jumps Core and complex movements Aquatic games	Technical-functional orientation
Pratt et al. [63]	Crawl, Backstroke and Breaststroke Push and Glide Sculling Tuck in water Submerging Floating Treading Water Aquatic Jump	Static and dynamic buoyancy Proprioception work Paddling Diving Impulses and slides Jumps Complex movements	Technical-functional orientation
Moura et al. [70]	Water entry Water orientation and adjustment at vertical position Breath control – immersion of the face and eye-opening Horizontal buoyancy Body position at ventral gliding Body position at dorsal gliding Body position at longitudinal rotation in gliding Body position at front and back somersaults Leg kicking with breath control at ventral body position, with flutter boards Without any flutter device Egg kicking with breath control at dorsal body position with flutter boards Without any flutter device Feet-first entry Head-first entry Autonomy in a deep pool (legs and arms displacement) Vertical buoyancy at deep water Deep water immersion	Static buoyancy Breathing Immersion Thrusts and slides Jumps Core and complex movements	Technical-functional orientation
Moreno-Murcia et al. [71]	Aquatic Utility Skills	Core displacements	Educational-curricular orientation
Pratt et al. [72]	Crawl, Backstroke and Breaststroke Floating Log Roll Submersion Jump Sculling Feet First, Sculling Headfirst, Treading Water Tuck	Static and dynamic buoyancy Proprioception work Paddling Diving Impulses and slides Jumps Core and complex movements	Educational-curricular orientation
Sundan et al. [64]	Water entry Swimming on the front Surface diving Float/rest Swimming on the back Water exit	Static buoyancy Dive Diving Core movements	Educational-curricular orientation
Shen et al. [73]	Aquatic breathing Buoyancy and balance Streamline glide Rotation and orientation Mini water polo Relay race Jumping in the water	Static and dynamic buoyancy Breathing Impulses and slides Jumps Handling, Throws and Catches Spins Complex movements	Technical-functional orientation

*Technical-functional orientation refers to studies primarily focused on the acquisition of functional aquatic competencies (e.g., swimming technique, water safety, balance, breathing control, or survival skills). Educational-curricular orientation refers to studies explicitly linking aquatic activities with broader educational aims or curriculum-related competencies within school contexts.

## Discussion

The objective of this systematic review was to analyse school swimming and water activity programmes, their methodological characteristics and the contingency plans developed at different educational stages within the school timetable. Overall, the evidence shows considerable heterogeneity in the design of the interventions, their educational alignment and the pedagogical approach adopted.

Since 2000, there has been a significant shift in the literature, with the mechanical learning of competitive styles being superseded by the concepts of “aquatic competence” and “aquatic literacy” [17]. This period also encompasses the transition towards more inclusive and diversified teaching in schools, allowing for the synthesis of the most rigorous evidence on how the aquatic environment contributes to the comprehensive development of students today.

The evidence analysed showed considerable heterogeneity in the design of the interventions and their alignment with national educational frameworks. Most of the studies reviewed were conducted mainly in countries such as Spain, along with other contexts such as Brazil, China, and England. Of these countries, only Spain and England had institutionalised teaching programmes such as “Swimming is Life” and the “National Swimming Curriculum”, aimed at systematic and competitive learning [34]. However, a legislative discrepancy was observed between the two: the British programme teaches swimming as a compulsory part of its core school syllabus [74], while in the Spanish system it is not compulsory and is subject to the request of schools.

With regard to the educational levels analysed, the results showed a clear preference for early childhood (3–5 years) and primary school (6–11 years) when examining swimming instruction for children in the literature [20,75]. This is probably because the period between 3 and 11 years of age is the optimal time to assess the development of core motor skills during childhood [27,76]. However, the review data also revealed, unlike the other stages, limited evidence in secondary education (6.2%) and a scarcity of interventions carried out as pupils progress through schooling [20,51].

The limited evidence identified in secondary education may reflect a combination of educational, organisational, and contextual factors. Adolescents often experience changes in motivation and interests, and some studies have reported perceptions of monotony or reduced engagement with school swimming activities [58]. However, structural barriers may also play an important role. Organising aquatic programmes for older students frequently requires greater logistical coordination, access to suitable facilities, transportation arrangements, and additional financial resources. These challenges may limit both the implementation of school-based aquatic programmes and the opportunities to evaluate them through research.

Consequently, the scarcity of studies in secondary education highlights the need for further investigation into how aquatic programmes can be sustainably integrated throughout the later stages of schooling. Expanding the evidence base is particularly important to understand how aquatic competence, water safety, and aquatic literacy can continue to develop beyond primary education.

Finally, limiting aquatic skills solely to childhood or to mastery of basic propulsion at these ages would interrupt the development of the swimming curriculum. This would not only weaken the continuity of the learning process, but would also contravene WHO safety guidelines. Therefore, adolescents require specific skills in addition to core skills in order to function successfully in variable aquatic environments (risk assessment and adaptability), as they are a high-risk group [3]. However, the lack of studies in secondary school reflects a disconnect between the physical education curriculum and the global need for advanced aquatic literacy, despite the fact that the benefits of swimming and its vital impact on human beings are well documented in the literature [77–80].

### Methodological quality and gaps in research

Analysis of the study design revealed a prevalence of observational and experimental methods, which contrasts with the scarcity of randomised controlled trials and longitudinal studies [51] on the importance of swimming in the development of core motor skills. Although most of the authors analysed obtained significant effects in the acquisition of aquatic competence through the descriptions of their interventions, the results of this review reveal a profound inconsistency in the configuration of school programmes.

The heterogeneity in the duration and frequency of the sessions constitutes a critical limitation that compromises the external validity of the proposals analysed. Although the findings of Jurak et al. [66] suggest that temporal dosing did not significantly alter the results, the lack of technical specifications in the works of Martins et al. [59], Pratt et al. [63] and Strašilová et al. [61] introduces a bias of indeterminacy. This omission of core variables not only erodes confidence in the reported effectiveness but also calls into question the replicability of the proposed models. Consequently, the scope of these findings should be interpreted with caution in the absence of a standardised operational framework. However, this notable methodological heterogeneity, with sessions ranging from 45 to 180 minutes, raises concerns from the perspective of motor learning theory, since under the principles of distributed practice [81], true aquatic literacy would require times that avoid neuromuscular fatigue and cognitive saturation [82]. This evidence suggests that, currently, organisational logistics prevail over the principles of motor consolidation. Given Ofsted’s [83] warning about the lack of time and poor quality of instruction in schools, a paradigm shift may be necessary. In this regard, Haga et al. [19] proposal to integrate weekly aquatic activity into the physical education curriculum (emulating the Icelandic system) could be presented as a possible structural solution. Therefore, implementing these programmes on a regular and sustained basis throughout the school years could become the only way to ensure real aquatic competence, based on skill transfer and long-term retention.

With regard to the assessment of aquatic competence, a wide variety of criteria was observed, which coincided with the findings of Minkels et al. [20] on the heterogeneity of aquatic learning methods in different contexts. However, the most notable evidence was the gap in the instruments used, with most studies (including recent publications such as those by Karatrantou et al. [67] and Moura et al. [69,70] continuing to use tools developed between the 1970s and 1990s. This disconnect between traditional instruments and current frameworks, such as aquatic literacy [33] or aquatic competence [17], once again highlights a methodological gap that urgently needs to be addressed.

Studies such as those by Stanković et al. [68] continue to use motor development tests focused strictly on product or technical efficiency [74,84]. This approach is insufficient in the face of contemporary standards that prioritise process, functional competence and risk management. There is also a lack of data on teacher preparation and the affective or cognitive dimensions of students, which is an intrinsic gap in the literature analysed. Only a minority of studies address variables such as motivation or self-confidence [67,71], which shows that current research continues to prioritise what is learned over how it is taught.

On the other hand, the discussion on material resources also reveals an unresolved pedagogical dichotomy, which adds to the aforementioned methodological shortcomings. The limited use of auxiliary objects in the studies reviewed seems to respond to historical controversies, which associated the material with an alteration of buoyancy [29] or a false sense of security [85]. On the contrary, contemporary evidence suggests that these resources are essential for improving motor performance and self confidence [86–88]. However, this discrepancy, coupled with external constraints such as the physical environment and cultural factors [89], highlights the need for a line of research that analyses the impact of resources from a holistic perspective. Taken together, these limitations highlight the urgent need to develop updated assessment protocols that are consistent with modern aquatic literacy frameworks and validated through comparative reliability studies.

### Pedagogical orientation of the programmes

The pedagogical orientation of the programmes analysed revealed considerable heterogeneity in the aquatic tasks implemented, through instructional, physical-sports and recreational approaches, aimed not only at developing basic swimming skills and improving technique, but also at strengthening the student’s autonomy and comprehensive aquatic competence. However, the findings highlight a lack of methodological justification linking these activities to any educational stage or structured progression in line with the school curriculum. This lack of transparency in the implementation of the programmes coincided with the opinions of Minkels et al. [20], who warned of deficiencies in the methodological quality of research related to swimming instruction. In contrast, only a minority of the studies analysed presented evidence based on institutional programmes, where activities were tailored to competency objectives and educational levels by age range.

The distinction identified between technical-functional and educational-curricular orientations suggests that aquatic activities in school settings are not always conceived as part of broader educational processes. Although many studies primarily focused on the development of functional aquatic competencies, only a limited number explicitly linked aquatic practice with educational aims and curriculum-related competencies associated with students’ holistic development. Importantly, this distinction was not based on the aquatic tasks themselves, since similar activities appeared across both categories, but rather on the pedagogical rationale and educational objectives attributed to aquatic practice within school contexts. This difference may partly reflect variations among national curricular frameworks, as some educational systems explicitly incorporate aquatic activities into Physical Education curricula, whereas others approach them mainly from a technical or safety-oriented perspective.

Regarding the prevalence of technical-functional approaches, the results revealed that most of the research (75%) maintained a focus on technique, propulsion, and swimming styles. This predominance shows that, despite experts’ efforts to orient school swimming towards competition and aquatic literacy [17,44,45], there remains a significant gap between scientific recommendations and actual teaching practice [90]. The reality in schools remains largely subordinate to the traditional performance models of institutions such as World Aquatics or national federation programmes, where the mechanical component prevails over the pedagogical, survival and autonomy in variable environments [91]. This pedagogical mismatch seems to be closely linked to a lack of shared vision between external entities and schools. Likewise, it has also been observed that the training of external instructors, often anchored in competitive frameworks, tends to displace the educational and global objectives of the curriculum, limiting the active participation of physical education teachers in planning and assessment. This displacement is particularly critical because physical education teachers possess the specific pedagogical and cross-curricular competencies needed to integrate aquatic activities into broader educational goals, a task that goes beyond the purely mechanical training usually provided by external instructors.

This disconnect could lead to a distancing from contemporary approaches to aquatic literacy, which advocate for comprehensive development that encompasses cognitive, affective, and physical dimensions [33]. Although the traditional technical model focuses on mastering specific styles, it is notable for its limited ability to enable students to achieve autonomous safety and adaptability to variable environments, which are principles advocated by the WHO. The analysis in this research suggests the need to move towards a comprehensive model of aquatic literacy. Criticism of technical-functional approaches in the school stage does not seek to question the effectiveness of the technique itself, but rather to point out its reductionist type. Indeed, while a technical-utility approach might be appropriate and sufficient for curricula strictly aimed at developing basic motor skills and water safety, it falls short when physical education pursues broader developmental goals. In such cases, the aquatic environment should be leveraged as a cross-curricular educational tool to stimulate cognitive development, including executive functions and decision-making. By focusing almost exclusively on the automation of movements, more traditional models omit the development of perceptual motor skills and the ability to make decisions in unforeseen situations in the aquatic environment. Therefore, students seeking functional autonomy in the water will acquire an incomplete education that prioritises mechanics over situational awareness. Furthermore, the results of the study coincide with the statement by Haga et al. [19], who highlight the scarcity of scientific literature and the lack of consensus on school aquatic standards. Likewise, only a small portion of the current evidence proposes an educational approach that is less sports-oriented and more integrated into the curriculum [92]. Therefore, the integration of aquatic literacy models into school syllabi would allow for the acquisition of technical skills to be reconciled with safety, inclusion, and lifelong enjoyment of the aquatic environment.

The findings of this review suggest that educational systems in which aquatic education remains optional may benefit from incorporating aquatic competence within national Physical Education curricula. Countries such as England and Norway provide examples of curriculum-integrated models that may contribute to a more systematic development of aquatic literacy, water safety, and lifelong participation in aquatic activities [76].

Therefore, educational policymakers should consider aligning national curricula with WHO recommendations on water safety literacy, promoting the progressive development of aquatic competence throughout compulsory schooling. Solutions such as standardising instructional time and competency objectives within official curricula could help prioritise swimming education and drowning prevention in schools. However, the feasibility of these proposals is hampered by a gap in scientific evidence, as research analysing the impact and integration of swimming into curriculum frameworks remains notably scarce in the literature [19]. Furthermore, methodological quality (control groups, objective measurements of acquired skills, and detailed descriptions of the programmes implemented) would be another limiting factor in drawing reliable conclusions [20].

### Limitations and future lines of research

One limitation of this review was the use of a ≥ 50% threshold for inclusion based on the STROBE and TREND checklists. While this criterion ensured broad inclusion of studies, it could be considered lenient compared to other reviews that applied stricter thresholds (e.g., ≥ 60%). Furthermore, although STROBE and TREND assess the completeness of reports, they do not assess methodological rigour or internal validity. To address this, the present review incorporated additional tools (NOS for observational studies and ROBINS-I for quasi-experimental designs) that assessed risk of bias. Additionally, the substantial heterogeneity observed among the included studies in terms of intervention duration, session frequency, pedagogical approaches, assessment procedures, and outcome measures limited the possibility of conducting direct comparisons between studies. Likewise, variations in the conceptualisation and operationalisation of constructs such as aquatic competence, aquatic literacy, and swimming skills may have affected the comparability and interpretation of the findings. However, no formal classification of the strength of evidence (e.g., GRADE) was applied, as the main objective was descriptive and exploratory, rather than recommendation-oriented.

Based on the results obtained, it is suggested that priority be given to analysing the divergence between swimming programmes integrated into the physical education curriculum and those managed by external entities. It would be relevant to examine how the professional profile of the instructor and the teaching styles used affect motor control and the development of general and specific motor skills. Likewise, future research should evaluate the degree of alignment of local practices with the international frameworks of the WHO, World Aquatics and the Aquatic Literacy Project, in order to standardise aquatic education and optimise student safety. Analysis of this gap would provide a rigorous scientific basis for future curriculum reforms. Finally, research is recommended to determine the ability of these programmes to facilitate learning transfer, and the extent to which a comprehensive motor skills foundation promotes the acquisition of advanced sports techniques in later stages of the training process.

## Conclusions

The findings of this review, together with the broader literature, support the importance of school-based aquatic education for promoting water safety and contributing to students’ physical, cognitive, and psychosocial development. However, substantial heterogeneity was identified across programmes, particularly regarding intervention duration, session frequency, pedagogical orientation, and assessment procedures.

Most of the available evidence focuses on early childhood and primary education, whereas research conducted in secondary education remains scarce. Furthermore, 75% of the included studies adopted a predominantly technical-functional orientation centred on swimming technique and skill acquisition, while only a limited number explicitly linked aquatic activities to educational objectives and curriculum-related competencies.

The methodological quality of the available evidence was generally moderate to low, and many studies relied on assessment instruments developed decades ago. Consequently, future research should prioritise more rigorous study designs, updated assessment tools, and greater curricular contextualisation of aquatic programmes.

Finally, the findings suggest that educational systems may benefit from integrating aquatic competence within Physical Education curricula through progressive and developmentally appropriate programmes. In this regard, restricting aquatic education primarily to childhood may hinder the continuity of learning and limit opportunities for the progressive development of competencies associated with aquatic literacy. This concern is particularly relevant in secondary education, where the scarcity of available evidence contrasts with the need for adolescents to acquire advanced competencies such as risk assessment, decision-making, and adaptability in variable aquatic environments, in line with WHO recommendations. Advancing towards comprehensive aquatic literacy models could facilitate the sustainable development of water safety, autonomy, inclusion, and lifelong engagement in aquatic environments.

### Practical recommendations

The findings of this review may provide practical guidance for Physical Education teachers and aquatic instructors when planning and implementing school-based aquatic programmes. Practical implementation should be based on a clear alignment between aquatic activities, swimming-related competencies, broader educational outcomes, and curriculum objectives. Activities such as floating, propulsion, water orientation, or problem-solving tasks in aquatic environments should not be understood solely as technical exercises aimed at acquiring aquatic skills, but also as opportunities to develop water safety, autonomy, decision-making, cooperation, self-confidence, and other competencies associated with students’ holistic development. These educational outcomes can, in turn, contribute to wider Physical Education curriculum goals related to motor competence, health promotion, personal development, social responsibility, and lifelong participation in physical activity. Establishing these explicit connections may facilitate the transition from predominantly technical-functional approaches towards more educational-curricular models of aquatic education, thereby strengthening the educational value of aquatic programmes within school contexts.

Early childhood education (age 3–5): A playful and exploratory pedagogical approach is recommended, prioritising familiarisation with the aquatic environment and overcoming fear through spontaneous play. Rigid technical teaching should be avoided in favour of developing core motor autonomy in the water and enjoying the aquatic experience.

Primary education (ages 6–12): A comprehensive model of aquatic competence should be adopted that goes beyond the traditional technical-sporting approach. Teaching should focus on the development of aquatic skills related to safety, survival and body control in the water. Swimming should be integrated into the Physical Education syllabus, focusing on global competency development through the aquatic environment, rather than being treated merely as an external or functional activity.

Secondary education (ages 12–16): Emphasis should be placed on promoting autonomy, health and participation in aquatic activities. In order to mitigate monotony and encourage motivation in students of this age, it is imperative to diversify the contents through the development of global aquatic skills and recreational activities. This strategy not only enriches the current experience, but also promotes the transfer of healthy habits, encouraging commitment to independent physical activity beyond the school environment.

## Supporting information

S1 File Search strategy. (PDF)
