# Supplementary material for: Analysis of swimming teaching programs, aquatic competencies and specific skills in school settings from early childhood to secondary education: A systematic review
Source: PLoS One. 2026 Jul 24;21(7):e0353477. doi: 10.1371/journal.pone.0353477 (PMC13399449; doi:10.1371/journal.pone.0353477)
Supplement: S1 File — (PDF) [file pone.0353477.s001.pdf]

S1. Search Strategy

1. **PUBMED**

Search fields: Title/Abstract [tiab] + MeSH terms
Date filter: "2000/01/01"[Date - Publication] : "2025/12/31"[Date - Publication]

("Swimming"[Mesh] OR "swimming"[tiab] OR "aquatic skills"[tiab] OR "water skills"[tiab] OR "aquatic competence"[tiab] OR "aquatic activities"[tiab] OR "aquatic exercises"[tiab]) AND ("Schools"[Mesh] OR "school"[tiab] OR "school education"[tiab] OR "physical education"[tiab] OR "high school"[tiab] OR "primary school"[tiab] OR "children education"[tiab]) AND ("Teaching"[Mesh] OR "teaching program"[tiab] OR "educational program"[tiab] OR "instruction"[tiab] OR "pedagogical approach"[tiab] OR "learning outcomes"[tiab] OR "skills development"[tiab]) AND ("english"[lang] OR "spanish"[lang]) AND ("2000/01/01"[Date - Publication]: "2025/12/31"[Date - Publication])

2. **Scopus**

Search fields: TITLE-ABS-KEY
Date filter: 2000 to 2025
Language filter: English, Spanish

(TITLE-ABS-KEY ("swimming" OR "aquatic skills" OR "water skills" OR "aquatic competence" OR "aquatic activities" OR "aquatic exercises")) AND (TITLE-ABS-KEY ("school" OR "school education" OR "physical education" OR "high school" OR "primary school" OR "children education")) AND (TITLE-ABS-KEY ("teaching program" OR "educational program" OR "instruction" OR "pedagogical approach" OR "learning outcomes" OR "skills development")) AND (PUBYEAR > 1999 AND PUBYEAR < 2026) AND (LANGUAGE (english) OR LANGUAGE (spanish))

3. **Web Of Science**

Search fields: TS= (Topic = title, abstract, keywords)
Date filter: 2000–2025
Language filter: English, Spanish

TS=("swimming" OR "aquatic skills" OR "water skills" OR "aquatic competence" OR "aquatic activities" OR "aquatic exercises") AND TS=("school" OR "school education" OR "physical education" OR "high school" OR "primary school" OR "children education")

AND TS=("teaching program" OR "educational program" OR "instruction" OR "pedagogical approach" OR "learning outcomes" OR "skills development")

Refined by: languages: (english or spanish) Timespan: 2000–2025

4. **SPORTDiscus**

Search fields: AB (abstract) and TI (title)
Limiters: Peer-reviewed, English/Spanish, 2000–2025

(TI (swimming OR "aquatic skills" OR "water skills" OR "aquatic competence" OR "aquatic activities" OR "aquatic exercises") OR AB (swimming OR "aquatic skills" OR "water skills" OR "aquatic competence" OR "aquatic activities" OR "aquatic exercises")) AND (TI (school OR "school education" OR "physical education" OR "high school" OR "primary school" OR "children education") OR AB (school OR "school education" OR "physical education" OR "high school" OR "primary school" OR "children education")) AND (TI ("teaching program" OR "educational program" OR instruction OR "pedagogical approach" OR "learning outcomes" OR "skills development") OR AB ("teaching program" OR "educational program" OR instruction OR "pedagogical approach" OR "learning outcomes" OR "skills development"))

Limiters: Published Date from: 20000101 to 20251231; Language: English, Spanish; Peer-reviewed: Yes
